# Azoreductase activity of dye-decolorizing bacteria isolated from the human gut microbiota

**DOI:** 10.1038/s41598-019-41894-8

**Published:** 2019-04-02

**Authors:** Sara A. Zahran, Marwa Ali-Tammam, Abdelgawad M. Hashem, Ramy K. Aziz, Amal E. Ali

**Affiliations:** 1 Department of Microbiology and Immunology, Faculty of Pharmaceutical Sciences and Pharmaceutical Industries, Future University in Egypt, New Cairo, 12311 Egypt; 2 Department of Microbiology and Immunology, Faculty of Pharmacy, The British University in Egypt, Shorouk City, Egypt; 3 Department of Microbiology and Immunology, Faculty of Pharmacy, Cairo University, Cairo, Egypt

## Abstract

The gut microbiota enriches the human gene pool and contributes to xenobiotic metabolism. Microbial azoreductases modulate the reduction of azo-bonds, activating produgs and azo polymer-coated dosage forms, or degrading food additives. Here, we aimed to screen the healthy human gut microbiota for food colorant-reducing activity and to characterize factors modulating it. Four representative isolates from screened fecal samples were identified as *E. coli* (AZO-Ec), *E. faecalis* (AZO-Ef), *E. avium* (AZO-Ev) and *B. cereus* (AZO-Bc). Both AZO-Ef and AZO-Ev decolorized amaranth aerobically and microaerophilically while AZO-Ec and AZO-Bc had higher aerobic reduction rates. The isolates varied in their activities against different dyes, and the azo-reduction activity mostly followed zero-order reaction kinetics, with a few exceptions. Additionally, the isolates had different pH dependence, e.g., AZO-Ec was not affected by pH variation while AZO-Bc exhibited variable degradation kinetics at different pH levels. Cell-free extracts showed NADH-dependent enzymatic activities 14–19 times higher than extracellular fractions. FMN did not affect the reducing activity of AZO-Ef cell-free extract, whereas AZO-Ec, AZO-Ev and AZO-Bc had significantly higher reduction rates in its presence (*P* values* = *0.02, 0.0001 and 0.02, respectively). Using Degenerate primers allowed the amplification of azoreductase genes, whose sequences were 98–99% similar to genes encoding FMN-dependent-NADH azoreductases.

## Introduction

The human body is inhabited by trillions of microbes that play essential roles in human health^1^. Most of these microbes reside in the human gut, mainly bacteria that belong to more than 1,000 species^2,3^. The gut microbiota enriches the human gene pool with millions of genes, about 150 times more than that found in the entire human genome, encoding enzymatic functions that complement and often surpass human metabolic capabilities^1^. This enables them to highly affect vital host biological processes such as biotransformation of orally administered natural products and xenobiotics^4–7^.

One such enzymatic activity performed by human gut microbes is the production of azoreductases, enzymes that degrade azodyes, extensively used in pharmaceuticals, food, cosmetic and textile industries. Azoreductases catalyze the reductive cleavage of azo bonds (–N=N–) to give colorless aromatic amines^6^. Azodyes used as food colorants are not harmful and their degradation products are not toxic. On the other hand, degradation of textile dyes give potential carcinogenic aromatic amines^8,9^. In pharmaceuticals, azodyes are used as basis for prodrugs and colon-targeted drug delivery systems. Prontosil, neoprontosil, sulfasalazine and olsalazine are well-known drugs, whose activation depends on intestinal bacteria^10,11^.

Azoreductase activity is expressed by several members of the human gut microbiota. Predominant bacterial genera with azoreductase activity found in the human intestinal tract include *Clostridium*, *Pseudomonas*, *Bacillus, Geobacillus, Lysinibacillus, Enterococcus*, *Eubacterium* and *Escherichia*^12^. Azoreductase activity of different species such as *Clostridium perfringens*, *Escherichia coli*, *Pseudomonas aeruginosa*, *Bacillus* sp., *Enterococcus faecalis* and *Enterococcus faecium* was studied^13–17^. On the other hand, azoreductase activity of other gut bacteria such as *E. avium*, *E. durans*, *E. gallinarum* and *E. hirae*^18^ is not documented.

Although azodyes were previously assumed to be only degraded under anaerobic conditions, recent studies described instances of aerobic degradation of azodyes, in which aerobic azoreductase enzymes are the major players^19^. In contrast to anaerobic reduction, aerobic reduction is not inhibited by molecular oxygen; thus, aerobically acting enzymes are termed oxygen-insensitive azoreductases. Aerobic reduction is considered more specific, since anaerobic reduction may be performed by low-molecular-weight redox mediators even in the absence of an anaerobically acting azoreductase enzyme^19^. Although oxygen-insensitive azoreductases have been previously reported, little information is available about their characteristics and substrate specificity; consequently we chose to focus on them in this study.

Based on their cofactor dependency, azoreductases were mainly classified into two major classes: flavin-free enzymes^20^, and flavin-dependent enzymes. The latter are further classified into NADH-dependent enzymes^21,22^, NADPH-dependent enzymes^23^, or enzymes dependent on both cofactors^24^. Of note, AzoA was the first identified aerobic azoreductase from human intestinal Gram-positive bacteria^24^. Besides oxygen sensitivity and cofactor dependency, two other main properties affecting azoreductase activity have been considered. These are the effect of pH and substrate specificity. The effects of these factors were best characterized through molecular cloning studies. Genes coding for oxygen-insensitive azoreductases were cloned from different organisms, such as *Bacillus* sp., *E. coli*, *E. faecalis* and *Pseudomonas* sp. proved to have specific substrates and optimum pH level^21,22,25–28^.

Although azoreductases are known to be involved in the biodegradation of food additives, and the activation of azo pro-drugs or azo polymer-coated dosage forms^29^, little is known about the rate and extent of azoreduction reactions of resident bacteria in the human gut microbiota. Here, we aimed to screen the gut microbiota of healthy humans for azoreductase activity on orally administered azodye-containing substances and characterize factors modulating this activity. Among the factors we studied are substrate specificity, effect of dye concentration and pH on enzymatic activity, cofactor requirements and the main cellular location of enzymatic activity within representative isolates from human fecal samples.

Finally, we amplified and sequenced azoreductase-coding genes from these isolates. Further characterization of azoreductases from the gut microbiota will help increase our knowledge about the fate of azodye-containing drugs or chemicals, and about differential human responses to them. This information will not only guide the development of more efficient drugs and dosage forms, but will also contribute to efforts for implementing microbiome testing in precision medicine and toxicology.

## Results

### Detection and identification of azoreductase-producing bacteria

**Figure 1:**
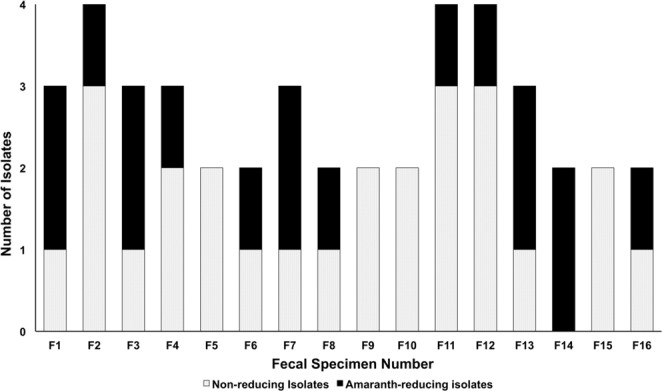
Number of morphologically distinct colonies. A stacked bar plot indicating the total number of isolated, morphologically distinct colonies and the number of amaranth-reducing colonies isolated from every stool specimen.

After aerobic incubation of the fecal dilutions for 24 h on BHIS agar supplemented with amaranth, 43 morphologically distinct bacterial colonies were recovered, 17 of which were surrounded by clear zones indicating their azoreductase activity **(**Fig. S1A**)**. Out of the 16 fecal samples, four did not show any azo-reducing colonies at aerobic conditions, while 12 had at least one active colony **(**Fig. 1**)**. This result was confirmed by the transfer of these colonies and their aerobic inoculation on fresh BHIS agar plates containing amaranth for single-colony isolation **(**Fig. S1B**)**.

These isolates were grouped according to their morphological characteristics and Gram reaction into three groups: Gram-positive cocci (11 isolates), Gram-positive bacilli (five isolates) and Gram-negative bacilli (one isolate) **(**Table 1**)**. Four representative bacterial isolates were selected for further studies, in which the effect of multiple factors on the azoreductase activity was tested. These isolates were selected according to their Amaranth-reducing activity and to be representative for the total number of every bacterial genus. They were identified as *E. coli* (named as AZO-Ec), *E. faecalis* (named as AZO-Ef), *E. avium* (named as AZO-Ev) and *B. cereus* (named as AZO-Bc) **(**Table 1**)**, and the sequences of their 16S rRNA genes were deposited in GenBank under accession numbers MG596978, MG596786, MH797010 and MG596976, respectively. Of note, AZO-Ev isolate was biochemically identified by API^®^ 20Strep as *Aerococcus viridans,* whereas its identification after 16S rRNA gene sequencing revealed 99% similarity to *Enterococcus avium* and 91% similarity to *Aerococcus viridans*   (Table 1). A previous study^30^ reported the biochemical relatedness of different streptococcal species (*A. viridans, S. uberis*, and *E. avium*) and that it was difficult to differentiate a *Globicatella sanguinis* isolate from these related streptococcal species. The close relation between these two species was confirmed by the high similarity percentage between them during the 16S rRNA gene identification of our isolate.

**Table 1:** Azoreductase-positive species identified in stool samples by API^®^ or VITEK^®^ identification system and 16S rRNA sequencing methods.

Isolate ID	Description	API identification (percentage of identification)	16S rRNA identification closest published sequence culture result of representative isolates (accession no., similarity)	Isolate Genbank accession number
K1	Gram-positive cocci (11 isolates)	*Aerococcus viridans (82%)*	*E. avium* (MH111545.1, 99%) OR *A.viridans* (KC699123.1, 91%) **Named as AZO-Ev**	MH797010
S1		*Aerococcus viridans (98%)*		
E2		*Aerococcus viridans (77.7%)*		
N2		*Enterococcus faecalis (89.1%)*		
N3		*Enterococcus faecium (69.4%)*	*E. faecalis* (KY438200.1, 99%) **Named as AZO-Ef**	MG596786
F1		*Lc. lactis lactis (90%)*		
NA2		*Lc. lactis lactis (95%)*		
DR1b		*Lc. lactis lactis (95.9%)*		
SH1		*Leuconostoc spp. (92.%)*		
B1		*Leuconostoc spp. (92.%)*		
NZ3		*Leuconostoc spp. (94.3%)*		
NZ1	Gram-positive bacilli (5 isolates)	*Bacillus cereus/thuringiensis (95%)*		
MS1		*Bacillus cereus/thuringiensis (94%)*	*B. cereus* (KU922458.1, 99%) **Named as AZO-Bc**	MG596976
NA1		*Bacillus cereus/thuringiensis (95%)*		
E1		*Brevibacillus choshinensis (93%)*		
DR1a		*Bacillus cereus/thuringiensis (92%)*		
SZ1	Gram-negative bacilli (1 isolate)	*Escherichia coli (99%)*	*E. coli* (CP021935.1. 99%) **Named as AZO-Ec**	MG596978

### Kinetics of decolorization of amaranth (20 µM) by whole cells under aerobic and microaerophilic conditions

**Figure 2:**
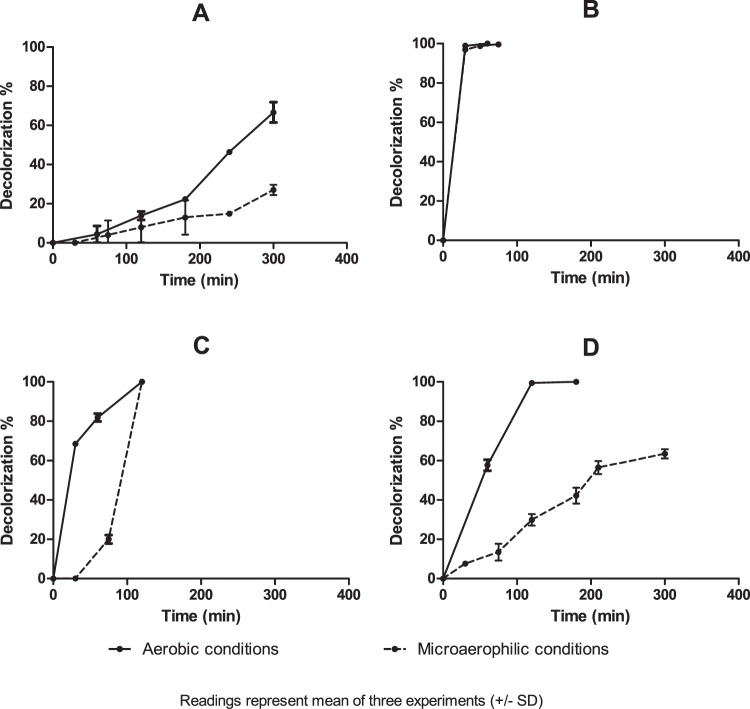
Percentage of amaranth (20 µM) decolorization by whole cells of the four selected isolates. (**A**) AZO-Ec, (**B**) AZO-Ef, (**C**) AZO-Ev and (**D**) AZO-Bc under aerobic and microaerophilic conditions. Readings represent the means of three experiments and error bars represent the standard deviation.

AZO-Ec and AZO-Bc showed different reduction rates of amaranth under both aerobic and microaerophilic conditions **(**Fig. 2**)**. AZO-Ec favored aerobic rather than microaerophilic decolorization: After 300 min, 66.67% of the dye was aerobically reduced in contrast to 26.06% reduction under microaerophilic conditions (Fig. 2A). Likewise, AZO-Bc showed higher reduction rate under aerobic conditions: Aerobically, almost complete decolorization (99.43%) was achieved after 120 min, while only 63.43% decolorization was achieved under microaerophilic conditions after 300 min (Fig. 2D). Of all four representative isolates, AZO-Ef was the most powerful reducing isolate, showing nearly complete decolorization after 30 min and with indifferent rates at both conditions (98.87% and 96.97% at aerobic and microaerophilic conditions, respectively) (Fig. 2B). Being an *Enterococcus*, AZO-Ev had a similar behavior to that of AZO-Ef. It was able to completely decolorize amaranth under both aerobic and microaerophilic conditions, but at a slower rate than that of AZO-Ef, as the dye was completely decolorized after 120 min under both conditions. Aerobically, AZO-Ev exhibited rapid initial decolorization rate, as decolorization reached 68.51% after 30 min. After 30 min, the reduction rate was slower. Under microaerophilic conditions, the reverse was observed, as no decolorization was detected during the first 30 min of contact but increased afterwards **(**Fig. 2C**)**.

### Decolorization of different azodyes by whole cells under aerobic conditions

After testing the kinetics of decolorization on amaranth (the dye that was used for screening) we expanded our analysis to a broader range of azodyes. As expected, the four isolates showed different percentages of decolorization with different azodyes. We found that, in general, a zero-order model best described the data fit for decolorization kinetics of AZO-Ec, AZO-Ef and AZO-Ev isolates at all tested substrate concentrations (r^2^ ≥ 0.8). However, when AZO-Ec was applied to amaranth and AZO-Ef was applied to sunset yellow, decolorization kinetics followed a first-order model at all tested concentrations (r^2^ ≥ 0.867 and 0.954, respectively). AZO-Bc enzymatic activity exhibited a zero-order kinetics model with different concentrations of amaranth and brilliant black (r^2^ ≥ 0.73 and 0.86, respectively). With sunset yellow and tartrazine, AZO-Bc followed a first-order model at low substrate concentrations (10 µM), but changed at higher concentrations (20 and 30 µM) to fit a zero-order model. The determination coefficients (r^2^) calculated for zero and first-order models are summarized in Supplementary Table 1.

**Figure 3:**
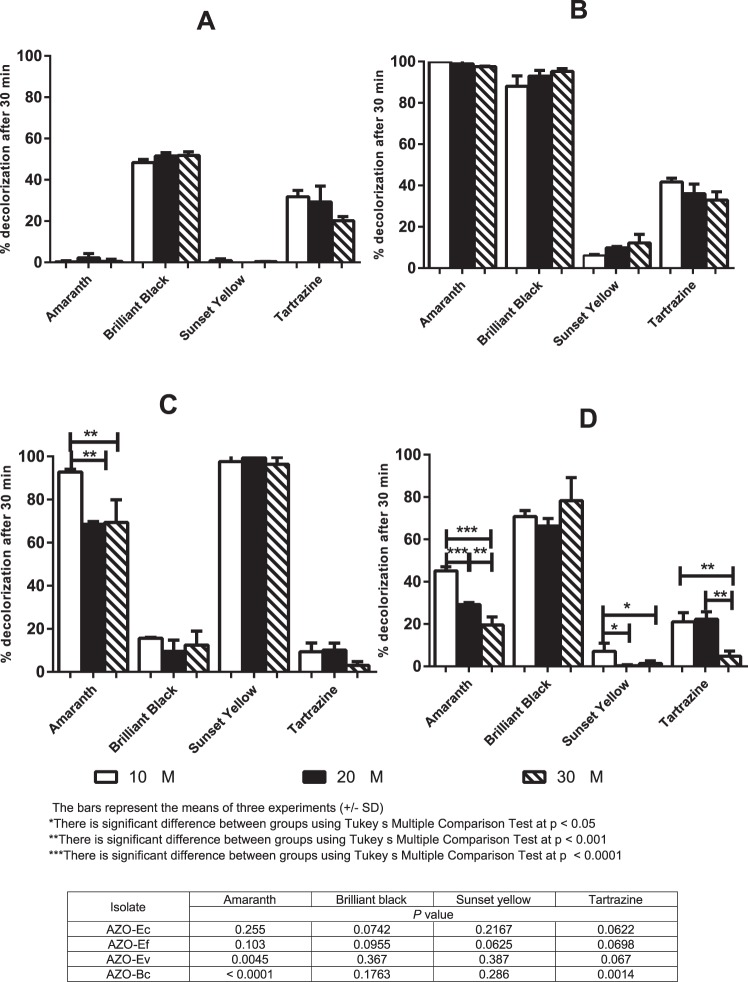
Percentage decolorization of four dyes after 30 min. Amaranth, brilliant black, sunset yellow and tartrazine were used at 10, 20 and 30 µM and their aerobic decolorization by: (**A**) AZO-Ec, (**B**) AZO-Ef, (**C**) AZO-Ev and (**D**) AZO-Bc was quantified. Bars represent the means of three experiments +/− standard deviation.

When we examined the percentage decolorization after 30 min of contact, brilliant black and tartrazine dyes were significantly reduced by all the isolates except AZO-Ev; amaranth was significantly reduced by AZO-Ef, AZO-Ev and AZO-Bc; while sunset yellow was only significantly reduced by AZO-Ev **(**Fig. 3**)**. With AZO-Ec, AZO-Ef and AZO-Ev changing the substrate concentration did not affect the percentage of decolorization detected after 30-min contact with any of the four tested dyes, except for a minor change in amaranth decolorization when its concentration was increased by two – three folds (*P = *0.0161) (Fig. 3A–C). On the other hand, the substrate concentration had some effects on the ability of AZO-Bc to decolorize three of the four dyes. Whereas varying concentrations of brilliant black did not significantly affect its reduction, increasing amaranth concentration (from 10 to 20 and to 30 µM) significantly decreased the percentage of decolorization after 30 min exposure (45.17, 29.18 and 19.53%, respectively, *P* value < 0.0001). With tartrazine, increasing the dye concentration also significantly reduced decolorization percentage (*P* value = 0.0014), but with no significant difference effect between 10 and 20 µM. Sunset yellow was significantly reduced by AZO-Bc after 30 min exposure only at the least used concentration (10 µM) (*P* = 0.0218), but higher concentrations did not show significant decolorization from nil (Fig. 3D). Growth of all isolates in the presence of the highest concentrations used (30 µM) was comparable in all cases, and no considerable difference in the generation time of either isolate was observed **(**Fig. S2**)**.

### Effect of pH on azoreductase activity

**Figure 4:**
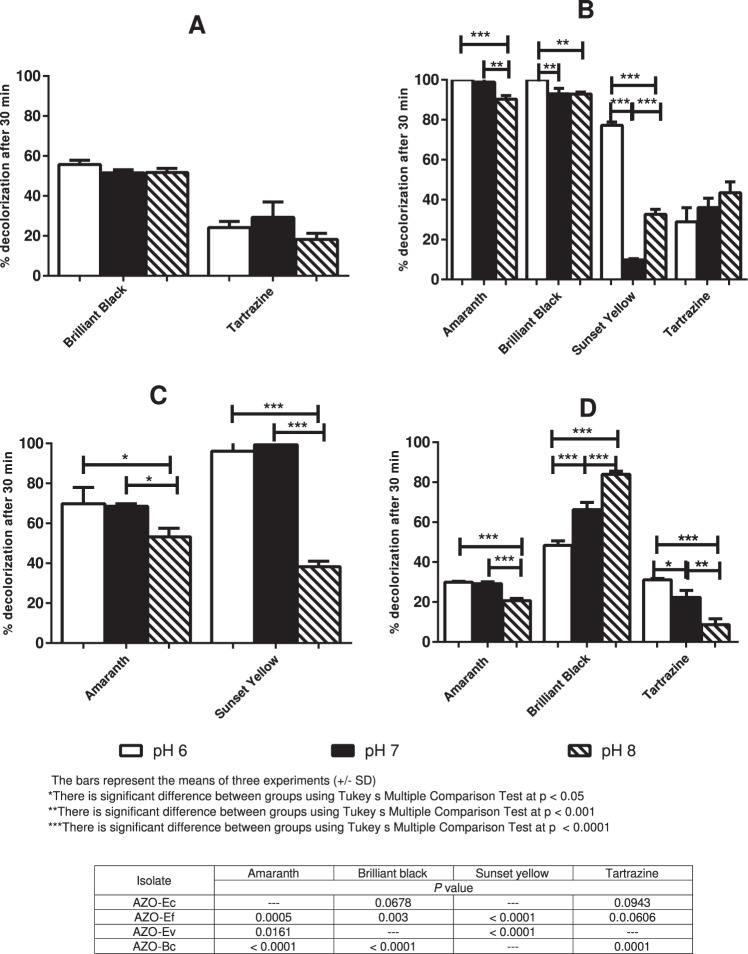
Effect of pH on dye decolorization by isolated bacteria. The effect of different pH values (6, 7 and 8) on the percentage of dye decolorization after 30 min was tested with different azodyes (used at 20 µM). (**A**) AZO-Ec, (**B**) AZO-Ef, (**C**) AZO-Ev and (**D**) AZO-Bc. Bars represent the means of three experiments ± standard deviation.

When the effect of pH on dye decolorization (20 µM) was tested on AZO-Ec, no significant difference in percentage of decolorization was observed after 30 min exposure (*P* value < 0.05) **(**Fig. 4A**)**. The two *Enterococcus* isolates favored either neutral or slightly acidic pH over pH 8. With AZO-Ef, most dyes were reduced at higher rates at pH 6 **(**Fig. 4B**)**. At pH 6, amaranth and brilliant black were fully decolorized by AZO-Ef while sunset yellow was decolorized to 77.19% (±1.63) of its original color. Tartrazine was the only dye equally reduced by AZO-Ef irrespective of the pH medium as the mean percentage decolorization did not significantly differ at different tested pH values (*P* = 0.0606) **(**Fig. 4B**)**. With the exception of brilliant black, AZO-Ev showed indifferent decolorization percentages between pH levels 6 and 7 for the other tested azodyes (*P* value < 0.05), while reduction percentages observed at pH 8 were significantly reduced (*P* value = 0.0161, 0.0034 and <0.0001 for amaranth, tartrazine and sunset yellow, respectively) **(**Fig. 4C**)**. Finally, with respect to the effect of pH, AZO-Bc behaved differently with every tested azodye. Amaranth reduction by AZO-Bc was generally modest, so the effect of pH changes was small yet statistically significant. A significant drop in percentage of amaranth decolorization was observed at pH 8 vs. either pH 6 or 7 (*P* < 0.0001). On the other hand, brilliant black and tartrazine reduction was highly influenced by pH variation. With tartrazine, the percentage decolorization at pH 8 was significantly lower than at pH 6 and at pH 7 with minor difference between pH 6 and 7 (*P* = 0.0001). The extent of brilliant black reduction significantly increased as the pH moved towards alkalinity, with all pH levels significantly different from each other (*P* < 0.0001), reaching a mean percentage of reduction of 83.92% at pH 8 **(**Fig. 4D**)**.

### Azoreductase assay of intracellular and extracellular fractions

**Figure 5:**
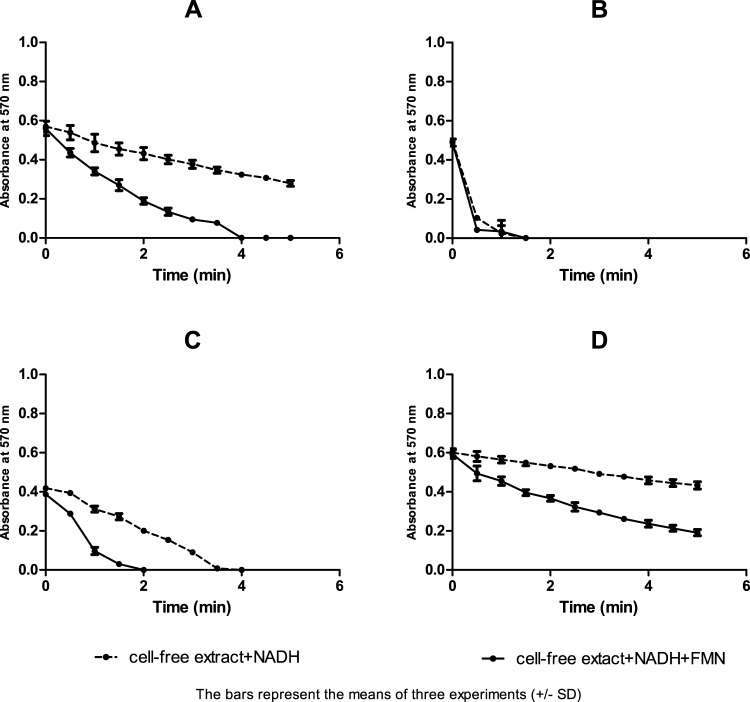
Kinetics of brilliant black reduction by cell fractions of the four isolates. Kinetics of brilliant black reduction by the cell fractions of: (**A**) AZO-Ec, (**B**) AZO-Ef, (**C**) AZO-Ev and (**D**) AZO-Bc, in the presence of NADH with or without FMN as cofactor. Readings represent the means of three experiments and error bars represent the standard deviation.

The detected enzymatic activity was mainly observed with the cell-free extracts of the four isolates, but not their supernatants. Such activity reached about 19 folds with the AZO-Ef isolate, 16 folds with AZO-Ev and 14 folds with AZO-Ec and AZO-Bc isolates, in comparison to extracellular fractions **(**Table 2**)**. The reducing activity of crude enzymes was only detected when NADH, but not NADPH, was used as an electron donor. Adding FMN as cofactor significantly improved the azodye-reducing activity of the cytosolic extracts of AZO-Ec, AZO-Ev and AZO-Bc, but not that of AZO-Ef when analysed using unpaired t-test (*P* < 0.05) **(**Table 2**) (**Fig. 5**)**.

**Table 2:** Enzyme activity in extracellular and intracellular fractions (U) against brilliant black (20 µM) with NADH as electron donor in the presence and absence of FMN.

Isolate	Enzyme activity in cell fractions (U) (mean ± SD)					
	Cell Extract			Extracellular		
	NADH + FMN	NADH	*P*-value	NADH + FMN	NADH	*P*-value
AZO-Ec	3.82 (±0.51)^b^	1.59 (±0.162)^b^	0.02	0.27 (±0.07)^b^	0.12 (±0.08)^b^	0.019
AZO-Ef	12.45 (±1.98)^a^	12.89 (±1.22)^a^	0.76	0.64 (±0.2)^b^	0.29 (±0.19)^b^	0.0215
AZO-Ev	7.93 (±0.58)^d^	2.85 (±0.26)^d^	0.0001	0.51 (±0.04)^b^	0.3 (±0.04)^b^	0.039
AZO-Bc	2.19 (±0.97)^b^	0.92 (±0.15)^b^	0.02	0.16 (±0.05)^c^	0.06 (±0.03)^c^	0.0027

*Standard deviation.

^a^There is no significant difference between the two treatments by using independent t- test (*P* ≥ 0.05).

^b^There is a significant difference between the two treatments by using independent t- test (*P* < 0.05).

^c^There is a significant difference between the two treatments by using independent t- test (*P* < 0.001).

^d^There is a significant difference between the two treatments by using independent t- test ( *P* < 0.0001).

### Molecular identification and sequencing of azoreductase-coding genes from the selected isolates

Species-specific degenerate primers were designed and used to confirm the presence of azoreductase-family genes in each of the four isolates. No sequences were available in GenBank for azoreductase genes in *E. avium*, so primers designed for both *E. faecalis* and *E. faecium* were used to amplify genes coding for azoreductase enzymes from AZO-Ev isolate. All reactions yielded specific products at the expected sizes, except for AZO-Ev isolate that did not produce amplified product with either primers. Overall, four amplified products were obtained: one from AZO-Ec; another from AZO-Ef; and two from AZO-Bc (using both Bc and Bth primers). All amplified products were sequenced. To confirm specificity and rule out cross-reactivity, we used each primer pair against all isolates, and no cross-reactivity was observed between different genera.

Sequence analysis confirmed the specificity of the PCR products, as all sequences expected azoreductase genes in NCBI non-redundant nucleotide database. Specifically, the amplified azoreductase gene from the AZO-Ec and AZO-Bc (using primers designed for *B. thuringiensis*) was 100% identical to the conserved *E coli* and *B. thuringiensis* FMN-dependent-NADH azoreductases. Likewise, amplification products from AZO-Bc (using primers designed for *B. cereus*) and AZO-Ef were 98–99% identical, on the DNA level, to FMN-dependent-NADH azoreductases encoded in the respective species. On the amino acid level, translation products of the four sequenced amplicons were identical to their respective homologs in NCBI RefSeq protein database. Nucleotide sequences of isolated genes were deposited in GenBank (Accession numbers MH151205, MH151206, MH151208 and MH151209) for PCR amplification products of Bc, Bth, Ec, and Efs primers, respectively.

## Discussion

Being the body site occupied with the largest microbiota and the main route by which xenobiotics enter our bodies, the gut represents the major niche for the metabolism and biotransformation of more than 60 drug substrates^10,11^. Many studies showed versatile enzymatic metabolic activities of the gut microbiota that are capable of metabolizing different classes of chemicals, increasing or decreasing their toxicity utilizing several bioactive enzymes^31–37^. Considering the significant implications of the microbial metabolism of azodyes on the human host and the limited studies characterizing the microbial species, genes and enzymes mediating azodye reduction, this study was launched to screen the gut microbiota for azoreductase activity. On initial screening, 17 isolates were obtained with potential azo-reducing activities. They belonged to the genera *Enterococcus*, *Aerococcus*, *Lactococcus*, *Leuconostoc*, *Bacillus*, and *Escherichia*, four of which were further identified as *E. faecalis*, *E. avium*, *B. thuringiensis/cereus*, and *E. coli*. These four isolates were the ones selected for further characterization and for determining factors affecting their azo-reducing activities. To date, only *E. faecalis* and *E. faecium* were reported to possess azoreductase activity with broad substrate specificity. In the current study, we investigated the azoreductase activity from a related gut mirobiota; *E. avium* whose growth was not affected by azodyes and was able to reduce the color of amaranth as a screening azodye.

Four different azodyes, commonly used as food colorants and that proved to have no adverse cytotoxic, carcinogenic or mutagenic effects^38–41^ were used as substrates for subsequent analyses. BHIS medium was chosen to simulate the nutrient-rich environment of the gastrointestinal tract. Isolates were allowed to reach log phase of growth before being in contact with their substrate as the continuous flow of nutrients in the gut of healthy individuals is thought to prevent resident gut microbes from reaching a stationary phase^42^.

In this study, we collected samples from healthy adult individuals to minimize diversity due to age, infection, antibiotic treatment, or surgical operation^43^. In spite of the high compositional diversity of the gut microbiota, it is not as diverse on the functional level because of its high degree of functional redundancy^44^. With regards to the azoreductase activity, several gut microbial species were reported to possess azoreductase activity including anaerobic bacteria^34^ and facultative anaerobes^24,42,45^. This commonness of potential azo-reducing enzymes may suggest the importance of this pathway for the bacteria as well as the entire intestinal ecosystem. Since our isolates were selected according to their activity under aerobic conditions, which has been less studied than anaerobic azo-reducing activity, it was not surprising they belonged to aerobic or facultative anaerobic species. Most of the detected reducing activities were either better under aerobic conditions (AZO-Ec and AZO-Bc) or showed no significant difference from microaerophilic reduction rates (AZO-Ef and AZO-Ev), suggesting possession of oxygen-insensitive azoreductases as reported for similar species^22,24,46^. All previous studies reported that aerobic treatment of azodyes is required for their complete degradation as biodegradation of the potentially carcinogenic aromatic amines that rise from azodye reduction only takes place under aerobic conditions and requires specified microorganisms^22,47^.

Previous studies reported that different bacteria vary in their substrate specificity and reduction rates of different azodyes, depending on variation in bacterial growth rates, enzyme productivity, enzyme activity or substrate structural diversity^34,48^. In fact, it was reported that variation in the decolorization activity is mainly affected by the structure and complexity of the dyes themselves, specifically on the nature and position of substituting groups in the aromatic rings^48^. Although all azodyes used in this study were polar sulfonated dyes, representing food colorants, most of them were efficiently decolorized by all isolates, as they all possess a hydroxyl group in their *ortho* position. This could be explained by a previous study suggesting that different periplasmic redox mediators could transfer reduction equivalents to externally located azodyes with no need to penetrate the cell membranes^49^, which was emphasized by Lui and coworkers^50^. Furthermore, brilliant black was observed to possess the highest reduction rate by all isolates; this may be due to the presence of four sulfonic acid substituents in its structure in comparison to two or three groups in other used dyes. The electron withdrawal effect of these sulfonic acid substituents reduced the electron density at the azo bond and thus facilitated its reductive cleavage^51^.

In this study, AZO-Ef had a broader substrate specificity, degrading all four dyes used, confirming previous data reported about the aerobic FMN-dependent azoreductase (AzoA) of *E. faecalis*^52,53^. In spite of being highly related to *E. faecalis*, AZO-Ev has different substrate specificity from AZO-Ef. This observation is not odd, as a previous study reported critical alterations in the active sites and thus the substrate specificity of two azoreductase enzymes isolated from *E. faecalis* and *E. faecium* in spite of being closely evolutionary linked^13^. AZO-Ev was the only isolate in this study that was able to totally reduce sunset yellow in only 30 min and efficiently decolorized amaranth (68.5%), while the two other dyes were not significantly reduced after 30 min of contact. AZO-Ec showed efficient decolorization of brilliant black (48%) and tartrazine (20%) (after 30 min incubation), while amaranth was less efficiently reduced and sunset yellow was not reduced by AZO-Ec up to 5 hours contact. These results do not reproduce a recent study that illustrated the ability of three *E. coli* isolates from human intestine to decolorize both tartrazine and sunset yellow up to nearly 70% within 5–6 hours, anaerobically^45^. A second study, however, purified two azoreductases from an *E. coli* isolate: one of them efficiently decolorized amaranth and tartrazine while the other one utilized them less efficiently^54^. Such discrepancies could reflect strain-specific variations, quite common in *E. coli*^55^. Lower reduction rates were observed with AZO-Bc and might be attributed to its slower growth rates compared to other isolates (Fig. S2).

For all tested isolates, with the exception of AZO-Bc, degradation rates were not affected by increasing substrate concentrations, which is in accordance with a previous study on *Bacillus* species^56^. AZO-Bc enzymatic activity exhibited different decolorization rates using different concentrations with two substrates (sunset yellow and tartrazine). Reaction rate changed from first to zero-order as the dye concentration increased, suggesting non-linear kinetics possibly resulting from saturation of enzyme-binding sites at a certain threshold, above which substrate concentration no longer affects reaction rate^57^. A study on *E. faecalis* showed that reduction rates of azodyes follows zero-order kinetics and that the reduction rate depends on bacterial growth rate rather than substrate concentration^58^. Some studies, using other dyes, showed that increasing substrate concentration could influence decolorization rates by imposing microbial toxicity^48^. However, microbial toxicity was excluded in our study, as tested dyes did not exhibit any inhibitory effect on bacterial growth (Fig. S2). AZO-Ev had a similar behavior to AZO-Ef, where in general different substrate concentrations did not influence reduction rates.

The pH values tested in this study were restricted to reachable levels inside the intestinal tract, i.e. pH 6,7 and 8^59^. Enzymatic activity of AZO-Ec was pH stable over the tested range, as was previously reported with a similar oxygen-insensitive azoreductase from an *E. coli* isolate^46^. AZO-Bc isolate, on the other hand, exhibited variable degradation kinetics at different pH values, showing highest degradation at pH 6 with amaranth and tartrazine, but an opposite trend with brilliant black (best reduced at pH 8). These results suggest pH-dependent dye-enzyme interactions, in agreement with a previous report, which extensively tested BTI10 *Bacillus* isolate against 16 different azodyes^60^. Both Enterococcus isolates showed reduced activity in slightly alkaline pH than pH levels 6 and 7.

Another finding in our study was that brilliant black was highly reduced by cell-free cytoplasmic extracts of all isolates in comparison to intact cells and extracellular fractions, suggesting that soluble cytoplasmic enzymes are involved in bacterial azoreductase activity^49^. However, the polar nature of the used sulfonated dyes suggests that dye reduction is mainly an extracellular process^61^. These two observations are not necessarily contradictory. A possible explanation for the discrepant observations is that redox mediators transfer reducing equivalents to polar dyes outside the cell. Another expalanation is that these redox mediators work through electron-transferring proteins located in the outer membrane^61^.

Detection of azoreductase activity in cell fractions additionally enabled us to determine electron donors and cofactors involved in their reaction. All tested isolates effectively utilized NADH, but not NADPH, as an electron donor, suggesting that they were all NADH dependent. NADH-dependency was previously reported for azoreductases purified from *E. faecalis*, *E. coli* and *B. cereus*^21,22,24,53,62–64^. As for cofactor dependency, addition of FMN did not prove to be crucial for reduction to start and proceed. However, our enzymes cannot be described as FMN-free, because a significant difference was observed between the azoreductase activity of AZO-Ev, AZO-Ec and AZO-Bc in presence of FMN than in its absence. This partial FMN dependency may have resulted from the presence of more than a cytosolic azoreductase enzyme or the presence of other non-specific xenobiotic reducers in the cell-free extract. Finally, AZO-Ef did not show a significant difference in azoreductase activity in presence or absence of FMN.

To accurately characterize the FMN dependency of our enzymes, activities of the purified apoenzymes should be assessed to control FMN concentration in the reaction. Given that the azoreductase genes detected in this study had near-identical sequences to FMN-dependent-NADH azoreductase genes from the respective species, we conclude that our enzymes most likely belong to FMN-dependent azoreductases. Moreover, all primers used in this study proved to be species-specific and showed no cross-reactivity with unrelated species. Of note, DNA from AZO-Bc yielded positive PCR products with primers designed to amplify azoreductase genes from both *B. cereus* and *B. thuringiensis*, but not *B. subtilis*. This cross-reactivity between *B. cereus* and *B. thuringiensis* emphasizes the close relationship between the two closely related species. Only AZO-Ev did not produce amplified products using any of the primers designed for related *Enterococcus* species, suggesting different sequences of genes coding for their azoreductase enzymes from those of other *Enterococcus* species available at the Genbank database. The same observation was also reported with AZO-Ef isolate; where it gave amplified products using Efs primers only but not Efm primers in spite of being designed for closely phylogenetically related bacterial species. These results could also be justified by the findings of Macwana *et al*.^13^ who reported that (AzoEF1) an azoreductase purified from *E. faecium* isolate showed only 67% amino acid sequence identity to AzoA from *E. faecalis*.

In conclusion, our work emphasizes the importance of the human gastrointestinal microbiota in the biodegradation of azodyes, especially those used as food colorants. The azoreductase activity of three representative isolates belonging to *E. coli*, *E. faecalis*, *E. avium* and *B. cereus*, was characterized. Our isolates can partially or fully degrade azodye under both aerobic and microaerophilic conditions, but with different reduction rates. They exhibit various substrate specificities but are not much affected by substrate concentration, and are mildly affected by reaction pH. All isolates seem to possess enzymes that require NADH as electron donor and are mostly FMN dependent, but further enzyme purification is recommended to ensure their FMN dependency. To the best of our knowledge, there are no published reports of the azoreductase activity of *E. avium*.

Ongoing work includes investigating the microbiome composition of the same fecal samples used in this study and from which the tested strains were isolated. Future studies should further analyse metagenomic sequencing for more systematic identification of azoreductase homologs encoded by the uncultured fractions of these samples.

## Materials and Methods

### Ethical considerations

All protocols were approved by the Ethical committee of Faculty of Pharmaceutical Sciences and Pharmaceutical Industries, Future University (Approval # REC-FPSPI-2/14). No invasive procedures were used for sample collections and no animal experiments were used. Informed consent was obtained from the healthy volunteers who donated stool specimens, and the nature of the study was clearly explained to them. All experiments were performed in accordance with relevant guidelines and regulations.

Fecal samples were collected from 16 healthy adult individuals (aged 20–40 years), with no previous history of gastrointestinal disease and who had not been prescribed antibiotics for at least three months prior to specimen collection. Azodyes used in this study are mainly food colors: amaranth, brilliant black, sunset yellow, and tartrazine, obtained from Sigma-Aldrich® (Taufkirchen, Germany). Structural information of dyes used is shown in Table 3.

**Table 3:** Structural information of azodyes used in this study.

Common name, FD&C no. and E no.	Chemical structure, Molecular formula and Molecular weight	Wavelength λmax (nm)
Amaranth, FD&C Red 2, E123,	(MF: C_20_H_11_N_2_Na_3_O_10_S_3_, MW: 604.47305)	512
Brilliant Black, Food Black 1 E151	(MF: C_28_H_17_N_5_Na_4_O_14_S_4_, MW: 867.68)	570
Sunset Yellow, FD&C Yellow 6, E110	(MF: C_16_H_10_N_2_Na_2_O_7_S_2_, MW: 452.37)	480
Tartrazine, FD&C Yellow 5, E102	(MF: C_16_H_9_N_4_Na_3_O_9_S_2_, MW: 534.36)	425

### Isolation and identification of azoreductase-producing bacteria

Fecal samples (1 g) were serially diluted in Brain Heart Infusion (BHI; LAB M®, UK.) supplemented per liter with: yeast extract (10 g) (LAB M®, UK), hemin (1 mg) (Sigma-Aldrich®, Germany), vitamin K1 (5 mg) (Sigma-Aldrich®, Germany), L-cysteine HCl (0·6 g) (Sigma-Aldrich®, Germany), NaCl (45 mg), NaHCO_3_ (0.4 g), K_2_HPO_4_ (75 mg), KH_2_PO_4_ (177 mg), (NH_4_)_2_SO_4_ (0.45 g), MgSO_4_.7H_2_O (0.945 g) and CaCl_2_.6H_2_O (15 mg). The pH was adjusted to 7.0^42^.

Dilutions were plated on BHIS agar containing amaranth with a final concentration of 80 mg/ml^34^ for screening of azoreductase producing bacteria. Plates were incubated overnight at 37 °C, under aerobic conditions, and were observed for clearance of the dye surrounding the colonies. Colonies were transferred to BHIS agar plates containing amaranth for single-colony isolation^42^.

All positive isolates were preliminarily identified according to their morphological characteristics, Gram reaction, catalase test, and API^®^ 20Strep for Gram-positive cocci, API^®^ 20E for Gram-negative bacilli, or VITEK^®^ BCL for Gram-positive bacilli. Four representative isolates were selected for characterization of their azoreductase activity. These isolates were selected according to their amaranth-reducing activity and to be representative for the total number of every group of isolates as follows: one Gram-negative bacillus, one Gram-positive bacillus, and two Gram-positive cocci. The selected isolates were further identified by 16S rRNA gene amplification and sequencing. Genomic DNA of the isolate was extracted by Genomic DNA extraction kit (Qiagen^®^, Germany). The 16S rRNA gene was amplified by the polymerase chain reaction using universal primers 27F (5′-AGAGTTTGATCCTGGCTCAG-3′) and 1492R (5′-GGTTACCTTGTTACGACTT-3′)^65,66^.

### Polymerase chain reactions (PCR) and DNA sequencing

PCR amplification process was carried according to the following steps: Initial denaturation of 94 °C 5 min, followed by 30 cycles of 94 °C 1 min, 55 °C 1 min, 72 °C 2 min and final extension of 72 °C 10 min. GoTaq polymerase (Promega, Madison, WI, USA) and its buffers were used in all reactions. Amplification products were analysed gel electrophoresis. When a positive product was confirmed, it was purified by QIAquick^®^ PCR Purification kit (Qiagen^®^, Germany) and sequenced (Clinilab, Cairo, Egypt). The quality of obtained sequence reads was checked and corrected using DNA Baser (www.DnaBaser.com). Best matches to these sequences were identified by NCBI BLAST, http://blast.ncbi.nlm.nih.gov ^67,68^, against the NCBI nucleotide (nr/nt) database or the 16S Ribosomal RNA sequences database.

### Kinetics of decolorization of azodyes by whole cells under aerobic and microaerophilic conditions

Overnight cultures of selected microorganisms grown in BHIS broth were transferred at 1:10 dilution to pre-warmed BHIS broth (with no dye) and incubated at 37 °C to an optical density of 0.4 at 600 nm, then immediately 10 ml were transferred to 40 ml BHIS supplemented with amaranth at a concentration of 20 µM, flasks were incubated at 37 °C under static aerobic conditions. For microaerophilic assay test tubes were incubated at 37 °C in an anaerobic jar (Oxoid^®^ Anaerobic Jar 3.5Ltr HP0011A) and microaerophilic conditions were created using AnaeroGenTM environment generating kits (Oxoid^®^ UK). At 30 min intervals for 5 hours, aliquots of 1 ml were withdrawn and centrifuged at 15000 × g for 5 min. The resulted supernatant was assayed spectrophotometrically to measure the decrease in absorbance at 512 nm. Decolorization activity was expressed in terms of percent decolorization according to the formula^17^:

1 $${\rm{Decolorization}}\,( \% )=\frac{{\rm{Initial}}\,{\rm{absorbance}}-{\rm{Observed}}\,\mathrm{absorbance}\,}{{\rm{Initial}}\,\mathrm{absorbance}\,}\times 100$$

where initial absorbance is the absorbance at zero time of incubation and observed absorbance is the absorbance after incubation time.

### Decolorization of different azodyes by the whole cell under aerobic conditions

The decolorization activities of the selected microorganisms were assessed on amaranth, brilliant black, sunset yellow and tartrazine at a concentration of 10, 20 and 30 µM. The percentage decolorization (calculated by Eq. 1) was monitored at time intervals as the decrease in absorbance at appropriate wavelength (as mentioned in Table 3) till reaching complete decolorization for a maximum of 5 hours.

To gain insight into the enzyme decolorization kinetics of the used substrates at different concentrations, we examined decolorization data according to the zero-order and first-order mathematical models. Q_t_ *vs*. t for the zero-order model and log (Q_0_ − Q_t_) *vs*. t for the first-order model, where Q_t_ is the percentage decolorization of the substrate at time t and Q_0_ is the substrate initial concentration.

The coefficients of determination (r^2^) were calculated by means of GraphPad Prism (La Jolla, CA, USA) software, and the highest value indicated the best fitting between the decolorization profile and mathematical equations^69^.

Percentages of decolorization were plotted after 30 min exposure (least time required to achieve complete decolorization) to analyze significance of different substrate concentrations on enzymatic activity. Toxicity of the tested azodyes at the highest used concentration (30 µM) was assessed by spectrophotometric monitoring of the bacterial 600 nm optical density at time intervals, in presence and absence of the dyes

### Effect of pH on azoreductase activity

The azoreductase activiy of the selected isolates was tested for different dyes at different pH levels (6, 7 and 8) under aerobic conditions, at fixed dyes concentration (20 μM). The pH of the medium was adjusted using 0.1 N HCl or 0.1 N NaOH^61^. Percentage decolorization was calculated as stated above and results were plotted after 30 min of exposure.

### Localization of azoreductase enzyme

Cells from 200 ml of overnight cultures of selected microorganisms were harvested by centrifugation at 6000 g for 10 min at 4 °C and the supernatant was collected for determination of extracellular enzyme activity. Cell pellets were washed twice with 50 mM Tris-HCL (pH 7.5) and suspended in 30 ml lysis buffer containing 0.2 M NaCl, 5 mM MgCl2, 1 mM PMSF, 1 mM DTT and lysozyme (1 mg/ml)^70^. Cells were disrupted by sonication using sonicator probe for 2 × 10 seconds pulses for 5 min interval at 60 Hz. at 4 ± 0.2 °C. Cellular debris and unbroken cells were removed by centrifugation at 15,000 g for 15 min at 4 °C and the supernatant was collected for determination of intracellular enzyme activity^42,70^.

### Azoreductase assay in intracellular and extracellular fractions and cofactor dependency

The reaction mixture contained 50 mM phosphate buffer (pH 7.2), 20 μM brilliant black, 0.5 mM NAD(P)H and 200 µl of the crude enzyme in a total reaction volume of 1 ml. The reaction was started by the addition of 10 μL of NAD(P)H, and was performed in the presence and absence of 20 µM FMN. Decrease in color absorbance was monitored in the first 5 min at 570 nm^71^. The concentration of brilliant black was determined from a calibration graph of known concentrations of the azodye dissolved in the assay buffer^42^. One unit of enzyme activity was defined as the amount of enzyme that catalyzes the reduction of 1 μmol of brilliant black per min^71^.

### Designing degenerate primers for azoreductase genes from the selected isolates

Protein sequences produced by different species of known azoreductase activity, belonging to the genera of the identified isolates (*Enterococcus*, *Escherichia* and *Bacillus*), were searched using TBLASTN from the NCBI. These sequences were retrieved to corresponding nucleotide sequences from the Genbank RefSeq database (https://www.ncbi.nlm.nih.gov/nucleotide)^72^. For each species, BLASTN^67^ was used to find all possible homologous genes from members of the same species. Then, multiple sequences of BLASTN results were used to identify conserved regions suitable for designing degenerate primers, and the primers were designed manually^63,73^. Overall, six pairs of degenerate primers were designed (Table 2), and their melting temperatures, hairpin and possibility of heterodimer formation were checked by the OligoAnalyzer3.1 software (https://eu.idtdna.com/calc/analyzer). Primer sequences, lengths and expected amplicon sizes are listed in Table 4.

**Table 4:** Primer pairs used in this study to screen for azoreductase genes.

Bacterial species of interest	Primers			Expected size of amplified DNA fragments (bp)
	Name	Sequence (5′-3′)	Length (bp)	
*E coli*	EcF	GGC GAA GAC AAA TTT **Y**AC ATC **R**GT AAT G	28	486
	EcR	CAG GGT ACT CTC AGT CTA ATC AGT TGT CC	29	
*Bacillus cereus*	BcF	ACA GCA AAT CCA AAT TCA GCA GAA GG	26	420
	BcR	CTC CTT CAG AAT A**W**A CGC CAC CTG TT	26	
*Bacillus subtilis*	BsF	GAA TTT TGC AGC **H**AG ATC TTT TGC TTC TTG	30	471
	BsR	CCT **Y**GG **M**AG AGA TAT GAT TAA CGG AAC ATT	30	
*Bacillus thuringiensis*	BthF	TTG CTA CTT TAG CTG CTT CTT CAA GAC CHG	30	579
	BthR	CCA GCG GAA CAA GCA GTT AGT GT**R** AA	26	
*Enterococcus faecium*	EfmF	AGT AGT CAA AGG TCA TCC CCT AAC TGC	27	567
	EfmR	TTT TGC ATC AAC TCA TCT GCA CGG TC	26	
*Enterococcus faecalis*	EfsF	CAA ACA TGC CGG AAA TCG ATG AAG AA	26	311
	EfsR	GAA GCC GCC ATT TGA TTG GAT GT	23	

Primer names, sequences, lengths and expected size of amplified DNA fragments (amplicons) are given for each primer. H = A + T + C, M = A + C, R = A + G, W = A + T and Y = C + T.

### Statistical analysis

All data from azoreductase assays were presented as the mean value of three independent measurements ± standard deviation. GraphPad Prism (La Jolla, CA, USA) software was used to calculate the standard error and other descriptive statistics as well as hypothesis testing to determine the level of significance. Significant differences between means were tested by unpaired t-test and one-way analysis of variance (ANOVA) with various post hoc tests: Tukey′s Multiple Comparison Test and Dunnett’s Multiple Comparison. Differences were considered significant at *P* < 0.05 unless otherwise stated.

## Supplementary information

Supplementary Data

## Data Availability

We declare that all materials and data are available for readers.
