# Supplementary material for: Azoreductase activity of dye-decolorizing bacteria isolated from the human gut microbiota
Source: Sci Rep. 2019 Apr 2;9:5508. doi: 10.1038/s41598-019-41894-8 (PMC6445285; doi:10.1038/s41598-019-41894-8)
Supplement: Supplementary file 1 — Supplementary Data [file 41598_2019_41894_MOESM1_ESM.pdf]

Azoreductase activity of dye-decolorizing bacteria isolated from the human gut microbiota

Sara A. Zahran, Marwa Ali-Tammam, Abdelgawad M. Hashem, Ramy K. Aziz, Amal E. Ali

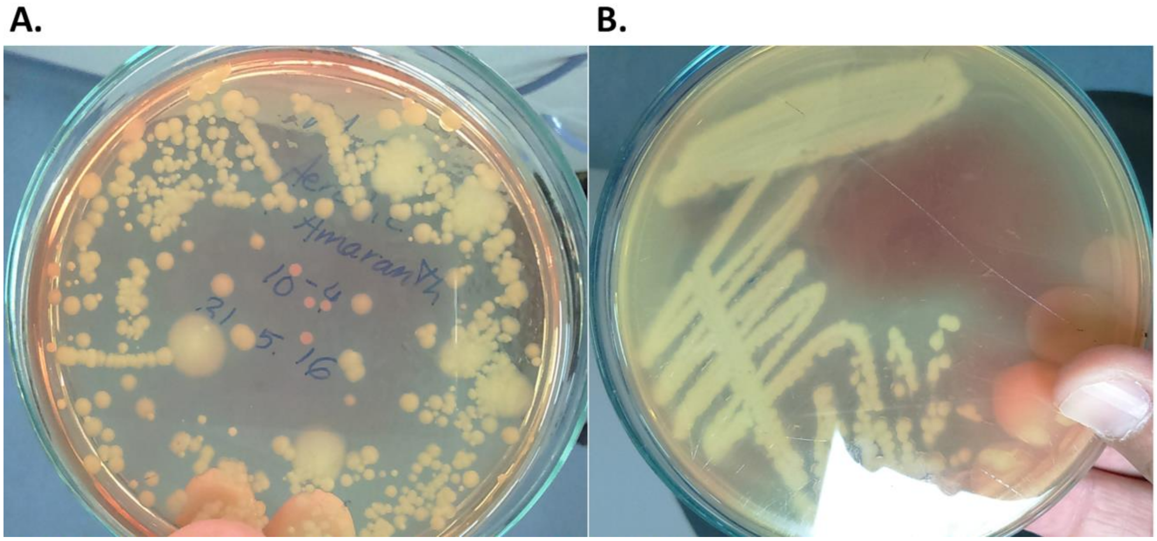

Figure-S1: Screening for azodye-reducing bacteria on BHIS agar supplemented with 20µM Amaranth. (A) Preliminary screening for azoreductase-producing bacteria from stool samples under aerobic conditions. (B) Single-colony isolation of colonies with promising activity in preliminary screens. Both cultures are on BHIS agar plates containing amaranth (20µM) and grown aerobically.

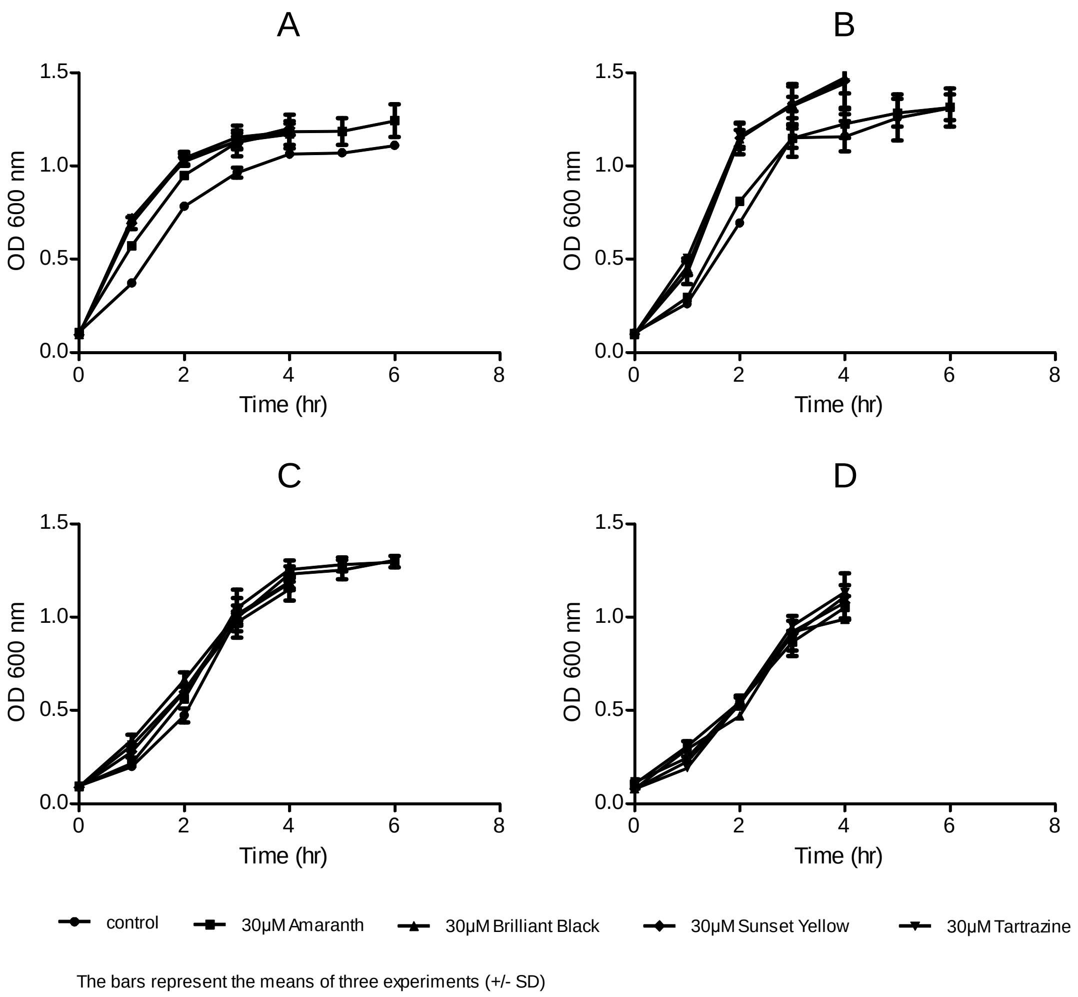

Figure-S2: Effect of azodyes on bacterial growth. Effect of used azodyes at a concentration of 30μM on the growth rates of the representative isolates: (A) AZO-Ec, (B) AZO-Ef, (C) AZO-Ev and (D) AZO-Bc. Readings represent the means of three experiments, and error bars represent the standard deviation.

Supplementary Table 1: Coefficient of determination (r^2) of azoreductase decolorization kinetics of the four selected isolates against different concentrations of different azodyes.

**Isolate**	**Substrate**	**Substrate Concentration (µM)**	**Zero-order r^2**	**First-order r^2**	**Isolate**	**Substrate**	**Substrate Concentration (µM)**	**Zero-order r^2**	**First-order r^2**
**AZO-Ev**	Amaranth	10	**0.997**	0.929	**AZO-Ef**	Amaranth	10	**0.981**	0.947
		20	**0.835**	0.729			20	**0.981**	0.947
		30	**0.800**	0.704			30	**0.981**	0.947
	Brilliant Black	10	**0.999**	0.946		Brilliant Black	10	**0.999**	0.950
		20	**0.990**	0.892			20	**0.999**	0.943
		30	**0.999**	0.928			30	**0.999**	0.935
	Sunset Yellow	10	**0.999**	0.969		Sunset Yellow	10	0.930	**0.959**
		20	**0.999**	0.969			20	0.839	**0.954**
		30	**0.999**	0.981			30	0.943	**0.973**
	Tartrazine	10	**0.983**	0.962		Tartrazine	10	**0.933**	0.872
		20	**0.954**	0.875			20	**0.955**	0.887
		30	**0.940**	0.854			30	**0.977**	0.939
**AZO-Ec**	Amaranth	10	0.864	**0.867**	**AZO-Bc**	Amaranth	10	**0.728**	0.693
		20	0.892	**0.957**			20	**0.992**	0.932
		30	0.789	**0.948**			30	**0.995**	0.976
	Brilliant Black	10	**0.999**	0.960		Brilliant Black	10	**0.904**	0.800
		20	**0.997**	0.926			20	**0.956**	0.860
		30	**0.995**	0.925			30	**0.861**	0.766
	Sunset Yellow	10	—	—		Sunset Yellow	10	0.928	**0.954**
		20	—	—			20	**0.926**	0.819
		30	—	—			30	**0.955**	0.871
	Tartrazine	10	**0.954**	0.905		Tartrazine	10	0.944	**0.979**
		20	**0.930**	0.924			20	**0.900**	0.833
		30	**0.821**	0.691			30	**0.976**	0.935
